# Nanoconfined anti-oxidizing RAFT nitroxide radical polymer for reduction of low-density lipoprotein oxidation and foam cell formation†

**DOI:** 10.1039/d1na00631b

**Published:** 2022-01-04

**Authors:** Suman Basak, Harshvardhan Ajay Khare, Paul J. Kempen, Nazila Kamaly, Kristoffer Almdal

**Affiliations:** Department of Health Technology, DTU Health Tech, Technical University of Denmark Kgs. Lyngby 2800 Denmark; Department of Clinical Physiology, Nuclear Medicine & PET and Cluster for Molecular Imaging, Rigshospitalet and University of Copenhagen Copenhagen 2200 Denmark; National Centre for Nano Fabrication and Characterization, DTU Nanolab, Technical University of Denmark Kgs. Lyngby 2800 Denmark; Department of Chemistry, Molecular Sciences Research Hub, Imperial College London London W12 0BZ UK; Department of Chemistry, Technical University of Denmark Kgs. Lyngby 2800 Denmark kral@dtu.dk

## Abstract

Atherosclerosis is a leading cause of death worldwide. Antioxidant therapy has been considered a promising treatment modality for atherosclerosis, since reactive oxygen species (ROS) play a major role in the pathogenesis of atherosclerosis. We developed ROS-scavenging antioxidant nanoparticles (NPs) that can serve as an effective therapy for atherosclerosis. The newly developed novel antioxidant ROS-eliminating NPs were synthesized *via* reversible addition–fragmentation chain-transfer (RAFT) polymerization and act as a superoxide dismutase (SOD) mimetic agent. SOD is an anti-ROS enzyme which is difficult to use for passive delivery due to its low half-life and stability. Copolymers were synthesized using different feed ratios of 2,2,6,6-tetramethyl-4-piperidyl methacrylate (PMA) and glycidyl methacrylate (GMA) monomers and an anti-ROS nitroxyl radical polymer was prepared *via* oxidation. The copolymer was further conjugated with a 6-aminofluorescein *via* a oxirane ring opening reaction for intracellular delivery in RAW 264.7 cells. The synthesized copolymers were blended to create NPs (∼150 nm size) in aqueous medium and highly stable up to three weeks. The NPs were shown to be taken up by macrophages and to be cytocompatible even at high dose levels (500 μg mL^−1^). Finally, the nitroxide NPs has been shown to inhibit foam cell formation in macrophages by decreasing internalization of oxidized low-density lipoproteins.

## Abstract 2

Atherosclerosis is a leading cause of death worldwide. Antiatheroscelerotic nitroxide nanoparticles effectively scavenge ROS and reduce the foam cell formation.

## Graphical abstract

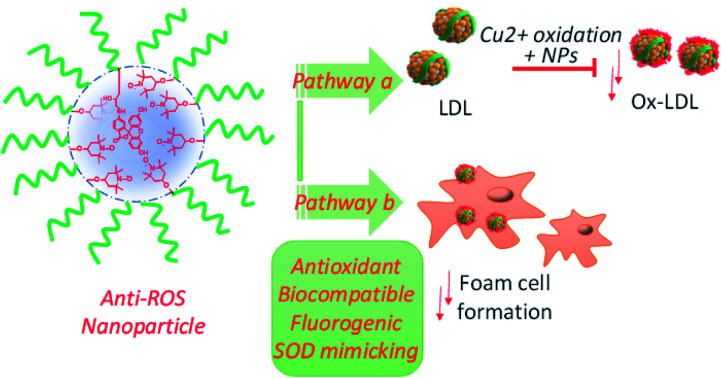

## Introduction

Atherosclerosis, a chronic inflammatory disease, is a major cause of coronary heart disease and stroke in humans.^1^ The disease is characterized by intimal plaques and cholesterol accumulation in the arterial walls.^2^ During atherogenesis, the primary process of inflammation is accompanied by oxidative stress, which is exemplified by the overproduction of reactive oxygen species (ROS) and oxidized low-density lipoprotein (ox-LDL). Hence an imbalance between radical production (reactive oxygen) and radical scavenging systems (the antioxidant defense system) is observed.^3^ High levels of ROS can induce oxidative stress that is closely associated with the pathogenesis of atherosclerosis. ROS can lead to NFκB activation, cell apoptosis, protein modification, and oxidative damage to other biomolecules.^4,5^ ROS also disrupts redox dependent signaling in the vessel wall to promote progress of atherosclerosis,^6,7^ involving signal transduction pathways,^8^ regulatory genes associated with vascular function,^7^ inflammatory components of atherosclerosis,^9^ and clearance of apoptotic cells by macrophages.^10^

Therefore, suppressing systemic oxidative stress, preventing vascular oxidative stress, and reducing ROS generation in plaques represent promising strategies for the treatment of atherosclerosis. In this respect, antioxidants have a crucial role in the prevention and treatment of atherosclerosis through different mechanisms. These include: the inhibition of low density lipoprotein (LDL) oxidation,^11^ the reduction of reactive oxygen species (ROS) generation,^12^ the inhibition of cytokine secretion, the prevention of atherosclerotic plaque formation and platelet aggregation, the preclusion of mononuclear cell infiltration, the prevention of endothelial dysfunction and vasodilation, the augmentation of nitric oxide (NO) bioavailability, the modulation of the expression of adhesion molecules such as vascular cell adhesion molecule-1 (VCAM-1) and intercellular adhesion molecule-1 (ICAM-1) on endothelial cells, and the suppression of foam cell formation.^13^ In this aspect, various types of antioxidants have been investigated,^14^ such as vitamins E and C,^15^ coenzyme Q,^16^ proanthocyanidin,^17^ 4-hydroxy-TEMPO,^18^ and NADPH oxidase.^19^ Although these preclinical studies have substantiated protective effects of antioxidants on atherosclerosis, clinical trials did not afford positive effects.^20^ Lewis *et al.* nicely demonstrated sugar based amphiphilic NPs to prevent oxidized lipid uptake and suppress scavenger receptor expression in macrophages.^21^ Annexin A1 actions were mimicked by Ac2-26 fragment containing NPs in reducing oxidative stress, promoting collagen buildup, and increasing the anti-inflammatory cytokine interleukin-10 and stabilize the plaque.^22^ In addition, recently developed 2,2,6,6-tetramethylpiperidine-*N*-oxyl (TEMPO) containing NPs significantly inhibited the development of atherosclerosis in ApoE−/− mice after i.v. delivery and the therapy afforded stabilized plaques with less cholesterol crystals and a smaller necrotic core.^23^ Thus, TEMPO and its derivatives are potentially powerful candidates for ROS scavenging.^24^ TEMPO decorated redox micelles have been used to suppress inflammation and treat drug-resistant in carcinoma cells.^25,26^

It appears that the NP based targeting strategies are effective and promising for molecular imaging and therapy of atherosclerosis.^27,28^ In atherosclerosis nano-delivery could be achieved in different ways such as: (i) nanoparticles that can target plaques by direct infiltration *via* the injured endothelium or the dysfunctional neovessels.^29,30^ (ii) Intravenous or intraperitoneal injection that can be endocytosed by circulating phagocytes, followed by translocation to atherosclerotic lesions.^31,32^ Therefore, nanoparticles serve as vehicles for targeted delivery of different therapeutics to atherosclerotic plaques and are a most promising way for next generation atherosclerotic drug delivery.^22,33,34^

**Fig. 1:**
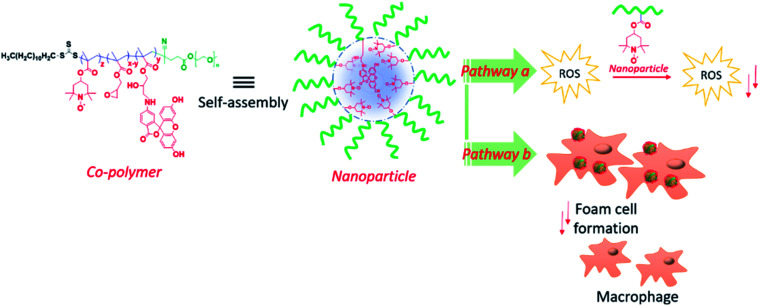
RAFT nitroxide NP design and application to the reduction of ROS species and macrophage foam cell. Our hypothesis is targeted on two different mechanistic pathways: (a) NPs quench free radicals and reduce the oxidation of LDL; (b) NPs induces the cellular uptake of ox-LDL and reduce the foam cell formation.

The nanogel NP based redox protein delivery plays an important role for the reduction of foam cell formation, as reported elsewhere.^35^ In redox protein therapy, it is challenging and difficult to protect the protein active site under physiological conditions. However, SOD enzyme delivery is well established and has several limitations, which include high production costs, short catalytic half-lives, limited active sites, and environmental sensitivity. We introduce a new type of RAFT mediated antioxidant NP for atherosclerosis therapy, which can mimic the SOD enzyme. The NPs are composed of diblock copolymers, consisting of polyethylene glycol together with PMA and GMA (Fig. 1). We hypothesize that the NPs will perform *via* two different mechanistic pathways: (a) NPs quench free radicals and reduce the oxidation of LDL by transition metal (*e.g.* Cu^2+^); (b) NPs induces the cellular uptake of ox-LDL and reduce the foam cell formation. Moreover, the RAFT nitroxide NPs have superior properties as they offer: (1) biocompatibility even at high dose level (500 μg mL^−1^), (2) efficiently mimicking SOD enzyme and very sensitive at low concentration limit, (3) NP stability in aqueous solution up to 3 weeks, (4) facile RAFT synthesis providing molecular weight control and monodispersity and easy NPs creation in aqueous medium, and (5) finally, softness, ∼100 nm PEGylated NPs potentially are useful as a passive delivery platform. Hydrophilic polyethylene glycol and hydrophobic GMA or 6-aminofluorescein (6-AF) renders the polymer amphiphilic and aids the self-assembly in aqueous solution. Moreover, polyethylene glycol improves the cytocompatibility of the NPs. The nitroxyl radical containing NPs facilitate the antioxidant ability and reduce the LDL oxidation. This NP could be easily up taken by the macrophages and inhibit ox-LDL induced foam cell formation that is closely related to the pathogenesis of atherosclerosis. Thus, these antioxidant NPs could be a promising therapy for atherosclerosis.

## Materials and methods

### Materials

**Fig. 2:**
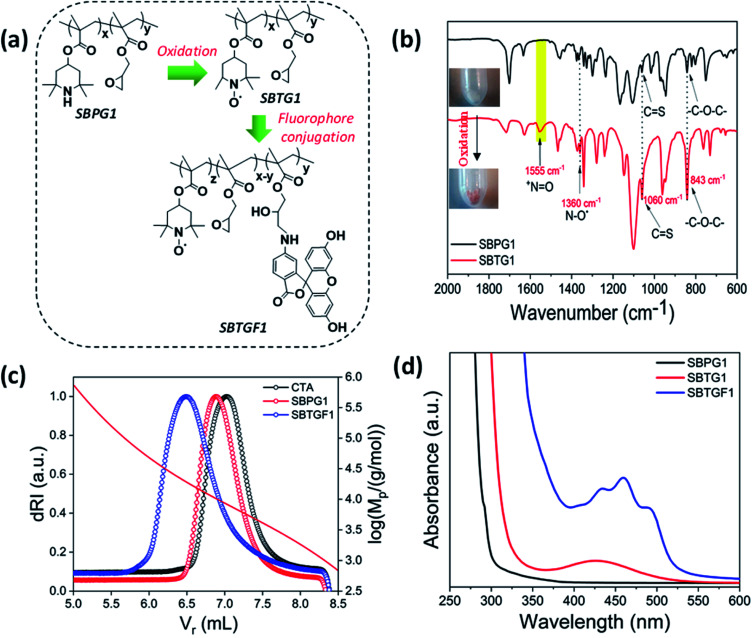
(a) Outline of RAFT nitroxide polymer synthesis. (b) FTIR spectra of copolymer before (black) and after oxidation (red). (c) SEC of macro-CTA, before and after fluorophore grafting polymer. For calibration curve – see Fig. SI2.† (d) UV-vis spectra of polymer before and after oxidation and after fluorophore (6-AF) grafting.

All the materials were used without any further purification. 2,2,6,6-Tetramethyl-4-piperidyl methacrylate (>98%; PMA) was purchased from TCI Chemicals Pvt. Ltd. Glycidyl methacrylate (≥98%; GMA) and poly(ethyleneglycol)methylether 4-cyano 4[(dodecylsulfanylthiocarbonyl)sulfanyl] pentanoate (PEGmacro-CTA; *M*_n_ = 10 kDa) were purchased from Sigma. We measured *M*_p_ = 8300 g mol^−1^ by SEC see Fig. 2 and *M*_n_ = 9.8 kg mol^−1^ by NMR. 6-Aminofluorescein (≥95%; 6-AF), 3-chloroperbenzoic acid (≥78%; mCPBA) and 2,2′-azobis(2-methylpropionitrile) (≥98%; AIBN) were purchased from Sigma. Human LDL (Sigma-Aldrich) and Oil Red O solution (0.5% in isopropanol, Sigma-Aldrich) were purchased from Sigma. Hoechst 33342 Solution (20 mM) (Thermo Scientific™), ProLong® Gold mount media (Thermo Fisher Scientific), Nunc™ MicroWell™ 96-Well Microplates – Thermo Fisher Scientific, Total Antioxidant Capacity Assay Kit (Sigma-Aldrich), Cell Counting Kit – 8 (Sigma-Aldrich), Dulbecco's Phosphate Buffered Saline (PBS) (Cell culture grade, Sigma-Aldrich), DMEM (Dulbecco's Modified Eagle's medium) (Sigma-Aldrich), chloroform-d 99.8 atom % D (≥99.8%, Sigma-Aldrich), dichloromethane ≥99.8% stabilized.

### Instruments and characterizations

#### ^1^H NMR spectroscopy

Varian Mercury 400 MHz Spectrometer. Sample dissolved in 550 μL CDCl_3_; Aldrich® ColorSpec® 400 MHz NMR tube. The data were analysed by using MestReNova software.

#### Size-exclusion chromatography (SEC)

Column: mixed D, 300 mm × 7.5 mm. 30 min of equilibration time before each injection of 250 μL of sample. Operating at 40 °C. Refractive index detector (DRI). Eluent: HPLC-grade DMF, 50 mM LiCl; flow rate: 0.25 mL min^−1^ 30 min equilibration time was used before each injection. Sample size: 250 μL. Calibration Polyethylene Glycol (PEG) standards (650 ≤ *M*/Da ≤ 9.42 × 10^5^). The chromatogram were analyzed using TriSec software.

#### UV-vis spectroscopy

Thermo Scientific Nanodrop 2000-C. Path length: 1 cm. 1 mL sample at room temperature. TEMPO radical shows absorbance at *λ*_max_: 460 nm and 6-AF *λ*_max_: 490 nm. 1 mg mL^−1^ solutions of SBPG, SBTG and SBTGF was used for absorbance measurement. Quartz cuvette in the wavelength range 200 to 800 nm.

#### FTIR spectroscopy

FTIR were recorded with freeze dried solid copolymer at room temperature (60 scans accumulated per spectrum) using Nicolet FTIR spectra equipped with Diamond ATR mode using Spectrum software.

#### DLS and zeta potential

Dynamic light scattering (DLS) was used to analyze the size and the charge of the nanoparticles using Malvern Zetasizer Nano-ZS. Backscatter detection was at 173°, hydrodynamic diameter was measured at 1 mg mL^−1^ sample concentration. Three replicas of 16 scans. Size and zeta potential measurement at 25 °C and 37 °C in deionized water (DI water) and PBS.

#### Cryo transmission electron microscopy (Cryo-TEM)

3 μL of nanoparticle solution was placed on a glow discharged lacy carbon 300 mesh copper TEM grid (Ted Pella, Inc.), blotted, and plunge frozen in liquid ethane using a FEI Vitrobot Mark IV. The Grids were imaged using a FEI Tecnai G2 T20 transmission electron microscope operated at 200 keV in low dose mode with a FEI High-Sensitive 4k × 4k Eagle camera located at the Core Facility for Integrated Microscopy, Faculty of Health and Medical Sciences, University of Copenhagen.

#### Spark® multimode microplate reader Tecan

The 96 well plate reader was used to measure the UV absorbances for different bio-assays.

#### Confocal microscopy

Cellular uptake of nanogels was analyzed by confocal microscopy. An upright laser-scanning microscope Zeiss LSM 710 was used with 63× magnification and the fluorescence nanoparticle intensity was collected at *λ*_ex_: 490 nm/*λ*_em_: 520 nm.

#### Synthesis of RAFT co-polymers, SBPG1–SBPG3

PMA (M1, 56.33 mg, 0.25 mmol), glycidyl methacrylate (M2, 35.53 mg, 0.25 mmol), PEG macro-CTA (50 mg, 0.005 mmol), AIBN (0.411 mg, 0.0025 mmol) and THF were sealed in a 10 mL reaction vial equipped with a magnetic stir bar. The vial was placed in an ice water bath followed by purging with N_2_ for 30 minutes. Next, the reaction vial was kept in a preheated polymerization block at 70 °C for 24 h. The reaction was quenched on an ice bath and exposing it to air. Unreacted monomer and CTA were removed by pouring the reaction mixture into a large excess of *n*-hexane, followed by centrifuging the resulting white cloudy dispersion at 3500 rpm for 15 minutes. The upper portion of *n*-hexane layer was carefully decanted. This was repeated six to eight times with a fresh batch of *n*-hexane each time. Then the purity of the resultant polymer was verified by ^1^H NMR spectroscopy and molecular weight was determined by SEC and NMR. The polymerization reaction was carried out at different feed ratios of (M1 + M2) : PEG macro-CTA : AIBN = 50/100/200/ : 1 : 0.5 (reported in ESI†).[M1 = PMA; M2 = glycidyl methacrylate]

#### Oxidation reaction of polymer

A solution of mCPBA (4.08 mmol) in 4 mL of anhydrous dichloromethane was made and SBPG1 (equivalent of 1.02 mmol M1-units) was dissolved in 4 : 1 (equiv.) ratio in anhydrous dichloromethane (DCM). The mCPBA solution was added drop-wise to the polymer solution and then allowed to stir at room temperature under nitrogen for 12 hours. The oxidized polymer was precipitated in hexane, filtered, and dried under reduced pressure overnight to get the final polymer (SBTG1). Note that the polymer changed from a white powder to an orange powder after oxidation. The successful oxidation of the nitroxide polymer was analyzed using UV-vis spectroscopy. The UV-vis spectroscopy was used as a complementary technique to measure the absorbance of nitroxyl group at 460 nm wavelength and to characterize the conversion.

#### 6-Aminofluorescein conjugation to the co-polymer

The copolymer SBTG1 (equivalent of 587 μmol of M2) and 6-aminofluorescein (1.46 mmol) were dissolved in 4 mL DMSO with DIPEA (2.34 mmol) and stirred for 24 h at 80 °C. Then, the mixture was precipitated in cold *n*-hexane five times. The precipitate was dialyzed (3500 kDa) against water : DMSO (3 : 1 (v/v)) at room temperature overnight to remove unreacted 6-aminofluorescein (6-AF). The pure 6-AF conjugated copolymer (SBTGF1) was freeze dried and obtained as an orange powder and used for further characterization.

#### Characterization of dye loading

The grafting of 6-AF from SBTGF1 copolymer was investigated in aqueous solution (mixed in 200 μL DMSO), and the grafting was confirmed using UV-spectroscopy.

#### Nanoparticles (NPs) preparation

For this, typically 3 mg of each polymer was dissolved in 1 mL of DMSO followed by dropwise addition of 5 mL of DI water with continuous stirring at 25 °C for 24 h. These polymer solutions were then transferred to the dialysis bag (MWCO = 3500) and dialyzed against large amounts of DI water for 24 h. The water was changed at regular intervals in order to remove DMSO and low molecular weight fragments completely, and facilitated the self-assembly of polymer chains.

#### Cell viability study

The cytotoxicity of the SBTG1 NPs was analyzed by the Cell Counting Kit 8 (CCK-8) from Sigma-Aldrich (Germany) according to the manufacturer's instructions.^36^ RAW 264.7 cells were cultivated in T-75 cell culture flasks using DMEM medium with 10% fetal bovine serum (FBS) supplement and 1% penicillin in passage-12. Healthy, RAW 264.7 cells were seeded in 96 well plate (5 × 10^3^ cells per well) in 200 μL of DMEM buffer with 10% FBS and 1% penicillin and allowed to grow for 24 h under incubation at 37 °C and 5% CO_2_. A set of different concentration of copolymer (SBTG1) was investigated for 24 h and 48 h and the absorbance was recorded at 450 nm background corrected for absorbance without the CCK-8 dye added using Tecan plate reader (Infinite pro-200, TECAN-reader). We note that the background correction accounts for less than 20% of the recorded absorbance. Measurements were done in triplicates and repeated three times.

#### Cell imaging and uptake study

Cellular imaging of SBTGF1 NPs were analyzed by confocal microscopy. For the cell uptake experiment, RAW 264.7 cells were seeded in the 12-well plate on top of the cover slip (after washing with ethanol and dried for 30 min) with a cell density of 10^5^ cells per mL for 24 h in an incubator at 37 °C, 5% CO_2_. Next, the cells were treated with LPS (100 ng mL^−1^) and IFN-γ (100 IU mL^−1^) for 24 h. After 24 h, media was removed (washed with PBS 3×) and cells were incubated with SBTGF1 NPs loaded with (50 μg mL^−1^ 6-AF) in DMEM media. For the control experiment, no SBTGF1 was considered as blank. At 24 h time intervals, the suspension was removed and wells were washed three times using PBS buffer. The diluted solution of cellMASK plasma membrane was stained for 10 minutes and washed with PBS. Fixed the sample with 4% formaldehyde solution (1 mL) and washed three times with PBS. Diluted Hoechst (33342) in PBS buffer with a concentration (1 : 5000, 1 mL per well), incubated for 15 minutes, 37 °C, 5% CO_2_ and washed with PBS buffer. Finally, cover slip was fixed on the glass slides using mounting medium (that can add favorable properties such as optimizing the refractive index to match that of glass, preventing photo-bleaching) and microscope Zeiss LSM 710 was used with 63× magnification to investigated the cellular uptake at the fluorescence intensity; *λ*_ex_: 490 nm/*λ*_em_: 520 nm.

#### Oil red O assay

Oil red O assay was carried out to examine whether treatment of SBTG1 nitroxide radical polymeric nanoparticle is effective to inhibit foam cell formation. RAW264.7 cells were stimulated with (100 ng mL^−1^) LPS and 100 IU mL^−1^ IFN-γ for 24 h then treated with 50 μg mL^−1^ ox-LDL for 24 h.^36^ Cells were preincubated with 10 μg mL^−1^ and 25 μg mL^−1^ SBTG1 NPs for 24 h. Control experiment was done without the addition of ox-LDL. Cell fixation was done by adding 10% formalin solution for 15 minutes and followed by stained with oil red O (ORO) and hematoxylin cocktail mixture. An optical microscope was used to observe the stained cells. For quantification of the oil droplet formation, PBS buffer added in each well was carefully discarded and air dried for 10 minutes. Then, stained cells in each wells were soaked in 2-propanol for 15 minutes to extract the dye. The lysis solution was transferred to a microplate to measure the absorbance 492 nm using a plate reader. The level of oil droplet formation was expressed as a value relative to that in the control group.

#### Antioxidant assay study

Total antioxidant capacity (TAC) of the synthesized polymeric NPs (SBPG1 and SBTG1) were analyzed by the total antioxidant capacity assay kit (Sigma-Aldrich, MAK187). Cu^2+^ reagent was diluted with 49 parts of assay diluent to prepare Cu^2+^ working solution. Trolox standard was prepared by reconstitution in 20 μL of DMSO with 980 μL of DI water to generate 1 mM concentration. A standard curve was prepared by a series of different diluted concentrations of Trolox sample by adding Cu^2+^ solution. A series of different concentrated NPs samples were investigated by dissolving the sample in DI water measuring UV-absorbance at 570 nm (*A*_570_ nm). Measurements were done in triplicates and repeated three times. Control was the SBPG1 polymer (non-oxidized) mixed with assay buffer. Amount of antioxidant was calculated using Trolox standard curve and total antioxidant concentration was considered using following equation:*S*_a_/*S*_v_ = concentration of antioxidant in each sample*S*_a_ = Trolox equivalent of unknown sample well (nmol) from standard curve. *S*_v_ = sample volume (μL) added into each well.

Furthermore, the anti-ROS efficacy of the RAFT nitroxide NPs was examined by malondialdehyde (MDA) lipid peroxidation assay (reported in ESI†).

#### MDA-lipid oxidation assay (TBARS assay)

The TBARS assay was performed according to the Lipid Peroxidation (MDA) Assay Kit (Sigma-Aldrich).^37^ Copper oxidized LDL was reconstituted in 20 μL PBS buffer and mixed with 500 μL of 42 mM sulfuric acid in a 1.5 mL Eppendorf tube. 125 μL of phosphotungstic acid was added and vortexed for 2 minutes. The sample and standards were incubated at room temperature for 15 minutes and centrifuged at 13 000*g* for 5 minutes. LDL was oxidized with 5 μM Cu^2+^ for 4 h at 37 °C and Cu^2+^ was removed by dialysis (with and without treated SBTG1 nitroxide NPs). The top layer was collected and mixed with 2 μL of BHT in 100 μL of deionized water and the final volume was adjusted to 200 μL. Finally, 600 μL of TBA solution was mixed together with the 200 μL analyte solution and incubated at 95 °C for 60 minutes and the UV absorbance of the solution measured at 532 nm.

## Results and discussion

### Design and synthesis of reversible addition fragmentation chain transfer (RAFT) copolymer

**Scheme 1:**
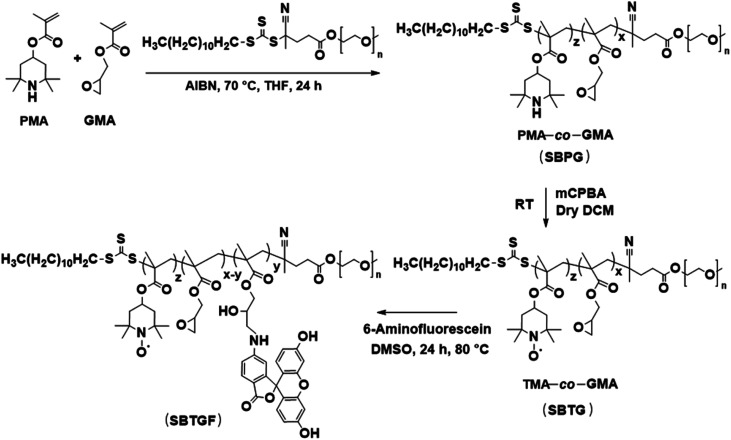
Synthesis of copolymer *via* RAFT polymerization, and grafting of 6-aminofluorescein *via* oxirane ring-opening reaction.

An amphiphilic copolymer was synthesized by the copolymerization of PMA (M1) and glycidyl methacrylate (M2) using poly(ethylene glycol) methyl ether 4-cyano-4-[(dodecylsulfanylthiocarbonyl)sulfanyl]pentanoate (PEG macro-CTA)as chain transfer agent as shown in Scheme 1. The linear diblock copolymers are designed to have a PEG hydrophilic block that displays biocompatibility, followed by a random sequence of free radical scavenging 2,2,6,6-tetramethylpiperidine-*N*-oxyl (TEMPO) and glycidyl methacrylate block that, at appropriate condition, will direct the self-assembly towards particle in aqueous medium (Fig. 1). Free radical scavenger 2,2,6,6-tetramethylpiperidine-*N*-oxyl (TEMPO) was chosen due to the known SOD mimicking activity and reduction of LDL oxidation.^23^ Copolymerization reactions of M1 and M2 were carried out using azobisisobutyronitrile (AIBN) as an initiator and PEG macro-CTA as chain transfer agent (CTA) in dry tetrahydrofuran (THF) at 70 °C *via* RAFT polymerization (Scheme 1).

For the synthesis of the RAFT nitroxide copolymer, PEGylated macro-CTA was used in order to produce a hydrophilic nanostructure, and ensure biocompatibility.^38^ After purification by precipitation in *n*-hexane, the copolymers were characterized by ^1^H NMR spectroscopy. The RAFT polymerization offer the synthesis of water soluble amphiphilic architectures with precise molecular weight and relatively narrow PDI.^39^ The copolymers were named as SBPG*x* where *x* represents the series of different feed ratios of M1 and M2 to the macro-CTA (SBPG1–SBPG3, Table 1). The macro-CTA to initiator ratio [macro-CTA]/[AIBN] = 1 : 0.5 was kept constant during all the polymerization reactions. After purification by precipitation in *n*-hexane, the copolymers were characterized by ^1^H NMR spectroscopy. The ^1^H NMR spectrum of the macro-CTA and SBPG1 are shown and discussed in the ESI.† The number-average molecular weight (*M*_n,SEC_) and molecular weight distribution (dispersity, *Đ*) values of the copolymers were evaluated by size-exclusion chromatography (SEC) in *N*,*N*-dimethylformamide (DMF) and results are summarized (Table 1).

**Table 1:** Results from the synthesis of copolymers and their size-exclusion chromatography (SEC) data in DMF medium

Polymer	[M1]/[M2]/[CTA]^a^	Conv.^b^ (%)	*M*_n_,_SEC_^c^ (g mol^−1^)	*M*_p,SEC_ c	*Đ* c	*M*_n,theo_ d (g mol^−1^)
SBPG1	50/50/1	30	8510	10 320	1.13	18 010
SBPG2	100/100/1	35	16 500	25 780	1.62	32 690
SBPG3	200/200/1	33	19 100	21 990	1.27	55 444

a [macro-CTA]/[AIBN] = 1 : 0.5 for all polymerization reactions. Polymerization time = 24 h.

b Determined gravimetrically.

c Measured using SEC analysis in DMF. *M*_p_ is the molecular weight based on PEG calibration at the peak of the elution curve.

d *M*_n,theo_ = ([M1 + M2]/[macro-CTA] × (*M*_w_ of M1 + *M*_w_ of M2) × conversion% + *M*_w_ of macro-CTA), where *M*_w_ = molecular weight of the components.

Differences between theoretical and experimental molecular weight can be attributed to the differences in hydrodynamic volume between SBPG*x* and the polyethylene glycol (PEG) calibration standard used (Fig. SI1†). Increase in the molecular weight with respect to the PEG macro-CTA confirmed the attachment of both M1 and M2 comonomers into the polymer chain (Fig. SI2†). SEC plots (see Fig. SI2†) of SBPG*x* polymers showed unimodal molecular weight distributions. The molecular weight of the copolymers increases with the increase of [monomer (M1) + (M2)]/[macro-CTA] ratio in the feed. Therefore, the number-average molecular weight (*M*_n,NMR_) of the copolymer (SBPG1) was evaluated by proton NMR analysis (see Fig. SI4, SI5, Tables SI1 and SI2†), and the *M*_n_,_NMR_ = 19.8 kg mol^−1^ for SBPG1 is comparable to the *M*_n,theo_. All copolymers solubility were tested in order to create NPs in aqueous medium. We concluded that the relatively hydrophobic nature of the side chain combined with the increased molecular weight lead to insolubility in aqueous medium for SBPG2 and SBPG3 (Fig. SI6†). Furthermore, the control of *Đ* is less satisfactory at high ratios. Thus, copolymer SBPG1 appear most attractive for the investigation of the potential to generate stable NPs useful in drug delivery.

Oxidation of the PMA block using mCPBA in dry DCM (Fig. 2a) was carried out at different time intervals (2 h, 4 h, 6 h, 12 h) and the reaction monitored using UV-spectrophotometry (Fig. SI7†). The conversion is linear in time and, thus the highest yield (%) was recovered after 12 h reaction. The oxidized polymer was precipitated in cold hexane and dried overnight to obtain an orange colour polymer named SBTG1 (copolymer containing nitroxide). The N–O˙ and +N=O stretching after oxidation (SBTG1) was examined by FTIR spectroscopy with a characteristic peak at 1360 cm^−1^ and 1555 cm^−1^, respectively (Fig. 2b).^40^ The epoxy group is intact with the polymer backbone and the asymmetric vibrational modes associated to the epoxy ring at 926 and 843 cm^−1^. The trithiocarbonate (C=S) unit at 1060 cm^−1^ is observed after oxidation and it is noted that the RAFT end functionality shown in (Scheme 1) is present after oxidation. The fluorophore (6-aminofluorescein, 6-AF) was grafted to SBTG1 polymer using oxirane ring-opening reaction, and was named SBTGF1 (copolymer containing both nitroxyl radical and fluorophore) (Scheme 1). The fluorophore (6-aminofluorescein, 6-AF) grafting was confirmed by SEC and UV-vis spectroscopy (Fig. 2c and d). We note that spectrophotometric quantification of both oxidation and fluorophore grafting is hampered by turbidity of the aqueous samples (see Fig. SI8†). The SEC shows considerable increase in the hydrodynamic volume on oxidation and the following fluorophore grafting. The *M*_p_-values of CTA, SBPG1, and SBTGF1 are 8300 g mol^−1^, 10 300 g mol^−1^ and 19 180 g mol^−1^, respectively.

### Nanoparticles (NPs) preparation and characterization

**Fig. 3:**
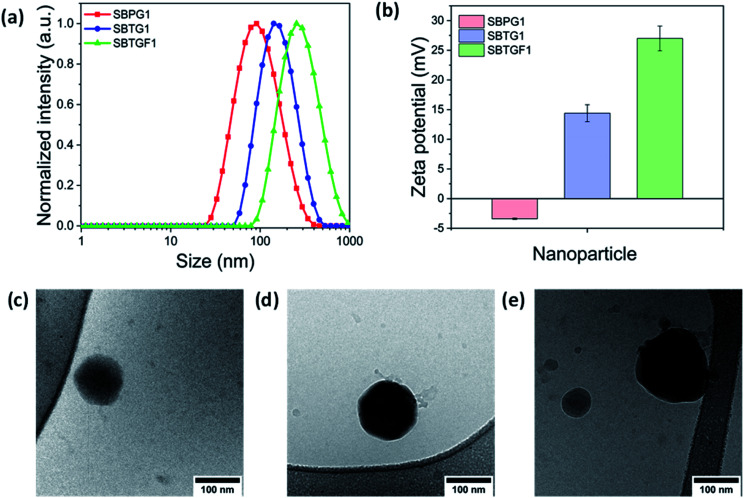
Dynamic Light Scattering (DLS) and TEM analysis of the Nanoparticles (NPs) (a) hydrodynamic size (nm). (b) Mean zeta potential (mV). (c)–(e) Cryo-TEM analysis of SBPG1, SBTG1 and SBTGF1 (left to right) respectively, scale bar = 100 nm.

Amphiphilic copolymers with densely grafted macromolecular architectures in selective solvents can possess a variety of morphologies such as spherical micelles,^41^ cylindrical nanostructures,^42^ multimolecular micelle^43^*etc.* However, these molecular assemblies are vastly dependent on the hydrophobicity/hydrophilicity (fb/fc) ratios of the copolymers.^44^ We have designed three different nanoparticles (NPs) named SBPG1, SBTG1 and SBTGF1 polymer based on the native, oxidized and fluorophore grafted structure. The nanoparticles were fabricated *via* the self-assembly of the copolymer by dialyzing their solutions in DMSO against deionized water (DI water). Because of the presence of long PEG side chains, the synthesized polymers form micelles in aqueous media with the M1/M2 random copolymer as core and the hydrophilic PEG as corona. First, the self-assembly behavior was investigated by dynamic light scattering (DLS) (Fig. 3a). The hydrodynamic diameter (*D*_h_) values were found to be 85 ± 5.2 (PDI = 0.24 ± 0.02), 120 ± 7.3 (PDI = 0.15 ± 0.03), 157 ± 2.1 nm (PDI = 0.32 ± 0.04) for SBPG1, SBTG1, and SBTGF1, respectively, with a monomodal size distribution and low PDI (Table 2). Furthermore, SBPG2 and SPBG3 show tendencies to form multimodal size distribution of the formed nanoparticles. This fact supports the choice of copolymer SBPG1 for the remainder of the study (see Fig. SI8a–c†).

**Table 2:** Size and zeta potential of nanoparticles (NPs)

Nanoparticle		Size (nm)	PDI	Zeta potential (mV)
SBPG1	In DI water	85 ± 5.2	0.24 ± 0.02	−3.4 ± 0.12
	In PBS	92 ± 1.2	0.37 ± 0.05	
SBTG1	In DI water	120 ± 7.3	0.15 ± 0.03	+14.4 ± 1.43
	In PBS	126 ± 6.7	0.34 ± 0.01	
SBTGF1	In DI water	157 ± 2.1	0.32 ± 0.04	+27 ± 2.09
	In PBS	159 ± 3.2	0.31 ± 0.03	

The significant increase in hydrodynamic size (*D*_h_), upon self-assembly in water of the three different SBPG*x* polymers ranging from 85–312 nm (Fig. SI8a–c†) as a function of [monomer (M1) + (M2)]/[macro-CTA] ratio in the feed is rationalized by the fact that the balance between hydrophilic (PEG) and hydrophobic (M1/M2 random copolymer) block is pushed towards a larger hydrophobic block. Further, the increasing size from SBPG1 to SBTGF1 micelles is due to stretching of the hydrophobic block in accordance with the size observed by SEC.

To gain more information on the morphology of the nanostructure of copolymers, cryo-TEM images were acquired. As shown in (Fig. 3c–e), SBPG1, SBTG1, and SBTGF1 copolymers self-assembled into spherical nanoparticles with an average size of 80 nm to 150 nm. The size increase from native nanoparticle (SBPG1) to fluorophore nanoparticle (SBTGF1) accommodates the chemical functionalization induced chain stretching of the micelle core forming block.

### Stability study

**Fig. 4:**
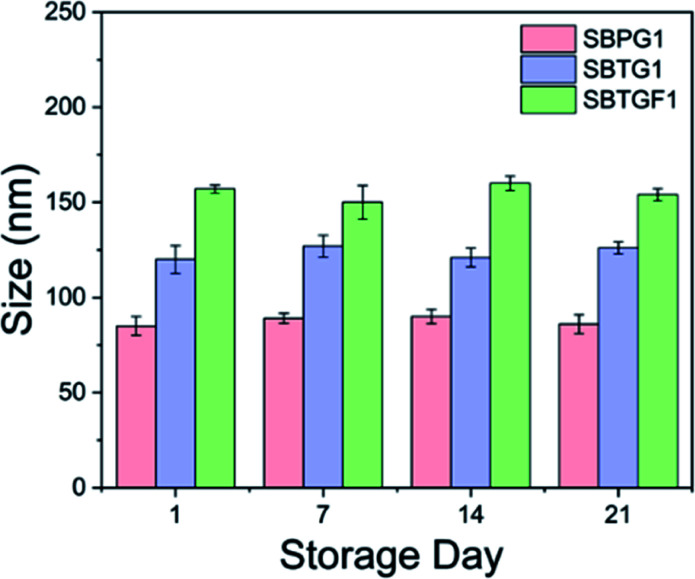
Storage stability of Nanoparticles (NPs) in deionized water (DI water) at 25 °C.

Nanoparticle (NP) stability was investigated using DLS measurements. An ideal nanoparticle should be stable in an aqueous medium for a sufficiently long time for storage purposes without aggregation. Furthermore, a higher colloidal stability is also correlated with the long circulation time *in vivo*. Thus, PEGylated core–shell nanoparticles show higher colloidal stability in aqueous media and are potentially useful for drug delivery application.^45^ The particle size was evaluated in PBS and deionized water (DI water) at 25 °C (Table 2) and the stability of the particle was studied in deionized water (DI water). It was observed that storage up to three weeks did not lead to nanoparticles aggregation and the size remains the same (Fig. 4). As shown in the Fig. 4, the nanoparticles are completely stable in DI water at 25 °C.

### Cellular internalization

**Fig. 5:**
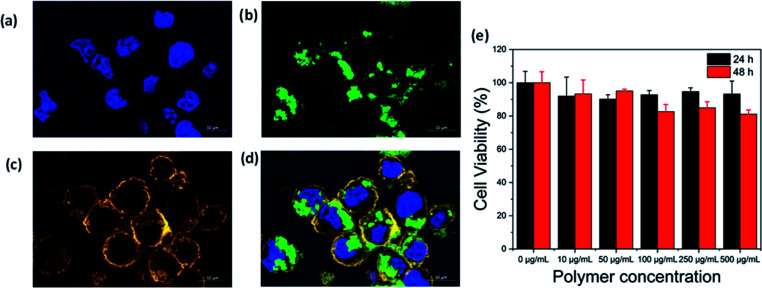
Intracellular delivery of SBTGF1 nanoparticles into RAW 264.7 cells by confocal laser scanning microscopy: (a) DAPI. (b) SBTGF1 nanoparticles. (c) Cell membrane stained. (d) Merged SBTGF1 nanoparticles with cell membrane stained and DAPI (scale bar 10 μm). (e) CCK-8 cell viability measurements of SBTG1 NPs after 24 h and 48 h incubation at 37 °C.

We investigated the cellular internalization of the 6-aminofluorescein (6-AF; *λ*_ex_: 490 nm/*λ*_em_: 520 nm) containing nanoparticles (SBTGF1). RAW 264.7 cells were seeded in the 12-well plate on top of the cover slip with a cell density of 10^5^ cells per mL for 24 h in an incubator at 37 °C, 5% CO_2_. The cells were treated with LPS (100 ng mL^−1^) and IFN-γ (100 IU mL^−1^) for 24 h. Next, RAW 264.7 cells were incubated with SBTGF1 NPs for 24 h at 37 °C. We observed respective signals at *λ*_em_: 457 nm, 520 nm and 590 nm, which corresponds to DAPI, nanoparticles and cell membrane staining, respectively (Fig. 5a–d). Efficient internalization was observed without causing any pernicious effect to the RAW 264.7 cells. Thus, the confocal study suggests the efficient uptake of the SBTGF1 nanoparticles in the perinuclear region of the RAW 264.7 cells.

### Cytotoxicity analysis

The cytocompatibility of the polymeric nanoparticles is an important factor for a drug delivery system.^46^ In this regards, we employed CCK-8 assay to determine the cytotoxicity of the nitroxyl radical containing nanoparticles (SBTG1) after incubation with the RAW 264.7 cell line. RAW 264.7 cells were exposed to various RAFT nitroxide polymer (SBTG1) NP concentrations. The results are shown in Fig. 5e, where more than 80% cell viability is observed with SBTG1 NPs for concentration between 0 and 500 μg mL^−1^ for 24 h and 48 h at 37 °C indicating biocompatibility. Hence, the low toxicity of the SBTG1 NPs toward the RAW 264.7 cells provides a direct indication that the newly developed nitroxyl containing copolymer can be administered in sufficiently high dose to function as an antioxidant nanodrug.

### Inhibition of foam cell formation by Oil red O (ORO) assay

Macrophage foam cell formation in the atherosclerotic plaque is a major hallmark of this disease and the process is mediated by the uptake of ox-LDL.^47^ The excessive uptake of ox-LDL by macrophages leads to their conversion into foam cells.

**Fig. 6:**
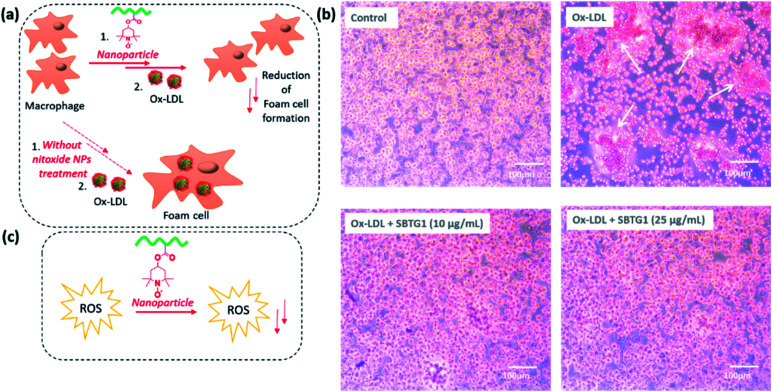
(a) Schematic illustration of anti-ROS, and reduction of foam cell formation effects of SBTG1 NPs. (b) Oil red assay in RAW 264.7 cell lines. Upper left panel: no ox-LDL; upper right panel: 50 μg mL^−1^ ox-LDL (24 h) – the white arrows point to lipid droplets – for an experiment that includes SBPG particles as well – see ESI Fig. S9†; lower left panel: 10 μg mL^−1^ SBTG1 followed by 50 μg mL^−1^ ox-LDL (24 h); lower right panel: 25 μg mL^−1^ SBTG1 followed by 50 μg mL^−1^ ox-LDL (24 h) (scale bar 100 μm). (c) Schematic illustration of anti ROS and free radical scavenging effects of SBTG1 NPs.

**Fig. 7:**
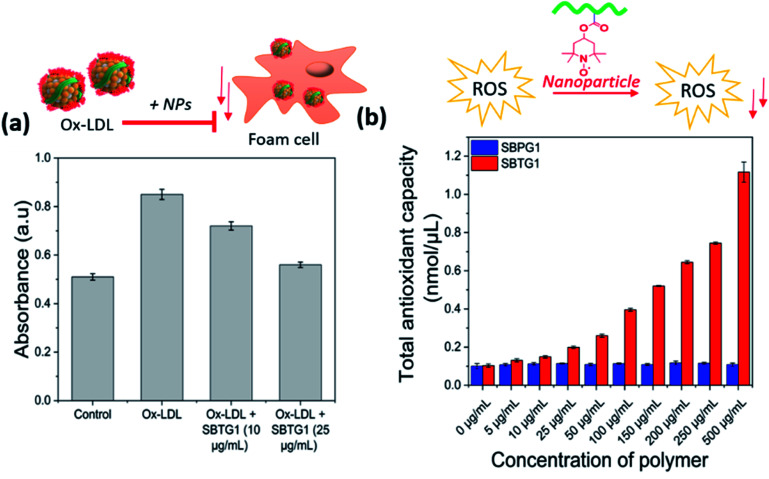
(a) Oil red O assay in RAW 264.7 cell lines, absorbance of lysis solution measures at 492 nm. (b) Cell free antioxidant capacity assay after exposure to SBTG1 and SBPG1 (control) NPs at different dose.

The LDL oxidation is mediated by superoxide (O_2_˙^−^) and hydroxyl radicals (˙OH) in the presence of transition metal ions (such as Cu^2+^).^48^ Furthermore, macrophages subsequently engulf oxidized low-density lipoprotein (ox-LDL) particles *via* scavenger receptors, mainly CD36 and scavenger receptor-A (SR-A), leading to the formation of foam cells. The hypothesis that treatment with nitroxide RAFT (SBTG1) nanoparticle is effective in inhibiting foam cell formation (see Fig. 6). Wang *et al.* reported that the reactive oxygen species scavenging nitroxide nanoparticles not only reduce the intracellular ROS production but also reduce the foam cell formation.^23^ It is reported that, nitro-oleic acid (NO_2_-OA) specifically interacts with CD36, thus limiting the binding and uptake of mLDL.^49^ Hence, antioxidant nano-therapy regulate the cellular ROS production and counteract the ox-LDL uptake.^50^ Therefore, we investigated the effect of SBTG1 NP treatment on the cellular uptake of ox-LDL in RAW264.7 cells. Anti-atherosclerosis effects of Mito-Tempol, an antioxidant, played a key role in autophagy restoration involving THP-1 macrophage-derived foam cells. Autophagy is restored by Mito-Tempol in ox-LDL loaded THP-1 macrophages *via* mTOR pathway and inhibits the ox-LDL induced foam cell formation.^51^ We examined whether treatment with SBTG1 NP effectively inhibit the ox-LDL uptake followed by foam cell formation. In this regards, we performed the oil red O (ORO) assay to identify the fat containing oil droplet by ORO staining. RAW 264.7 macrophages treated with 50 μg mL^−1^ ox-LDL for 24 h showed considerable intracellular lipid droplet formation (Fig. 6b, denoted by arrows on top right panel) and significant macrophage foam cell formation, as represented by staining with Oil Red O (ORO) (Fig. 6b). Pre-treatment with SBTG1 NP at either 10 or 25 μg mL^−1^ significantly reduced (at 10 μg mL^−1^) the formation of foam cells and notably suppressed (at 25 μg mL^−1^) foam cell formation. Control experiment was done without addition of ox-LDL. Quantification of intra-cellularly deposited ORO supported this microscopic observation (Fig. 6b). UV-vis spectra of the lysis solution provided the quantitative estimation of the oil droplet formation (Fig. 7a). Thus, these results demonstrated that SBTG1 NPs could attenuate the formation of foam cells from macrophages by reducing cellular internalization of ox-LDL almost to the level seen without ox-LDL exposure. A control experiment shows that nanoparticles without the nitroxy function does not induce the antioxidant function of the nanoparticles (see Fig. SI10†).

### Antioxidant effect of RAFT nitroxide nanoparticles

Oxidation of low-density lipoprotein (ox-LDL) is a key step in atherosclerosis, and transition metal ions (*i.e.* Cu^2+^) are implicated in this process. Interestingly, copper (Cu^2+^) levels are significantly increased in atherosclerotic lesions, compared with healthy, arterial walls, and promotes lipid peroxidation. A catalytic quantity of copper (Cu^2+^) together with superoxide (O_2_˙^−^) can stimulate LDL oxidation.^52^ (Cu^2+^) catalyzed breakdown of LOOH to reactive peroxyl (LOO˙) and alkoxyl (LO˙) radicals according to the reaction (1) & (2). Nitroxide radicals are effective to inhibit lipid peroxidation and scavenge free radicals. Nitroxide radicals have been found to protect cells against a variety of agents which cause oxidative stress,^53^ including superoxide,^54^ hydrogen peroxide,^54^ and oxidation of reduced metal ions, such as Cu(i) and Fe(ii) a reaction that promote oxidation.^55^

Therefore, we hypothesized that our RAFT nitroxide NPs could efficiently scavenge these free radicals and thereby reduce the LDL oxidation.

1 LOOH + Cu(2+) = LOO˙ + Cu(1+)

2 Cu(1+) + LOOH = LO˙ + OH(−) + Cu(2+)

The measurement of the total oxidant status (TOS)^56^ accurately reflects the oxidative stress markers of atherosclerotic environment. Nitric oxide (NO) acts as a free radical scavenger and contributes to host defence against oxidation. MDA is a reliable and commonly used marker of overall lipid peroxidation levels and the presence of oxidative stress.^57^ However, nitric oxide has been shown to reduce the lipid peroxidation by quenching the ROS.^58^ Therefore, this study aimed to determine oxidative stress levels that is associated with the overproduced ROS. The anti-ROS effectiveness of the RAFT nitroxide NPs was shown by the reduction of MDA formation (Fig SI10†) the antioxidant activity of SBTG1 NPs was evaluated using TAC (total antioxidant capacity assay),^59^ where the Cu^2+^/Cu^+^ system is used for antioxidant capacity quantification determined by UV spectrophotometry using Trolox standard. SBPG1 was considered as a control. SBPG1 and or, SBTG1 NPs were treated with different concentrations, and at higher concentration, the total antioxidant capacity (nM μL^−1^) increased (Fig. 7b). At 50 μg mL^−1^ the SBTG1 NPs showed almost double activity to the SBPG1 NPs. Thus, these results demonstrated that SBTG1 NPs at lower dose (50 μg mL^−1^) can serve as a potential antioxidant to reduce over expressed ROS and protect from oxidative damage.

## Conclusion

In summary, we report the development of stable antioxidant and anti-ROS SOD mimicking polymeric nanoparticle. The nitroxyl radical containing NPs effectively show antioxidant property. The NPs could also significantly decrease cellular uptake of ox-LDL and thereby reduce foam cell formation in macrophages. The nanoparticles are efficiently internalized into the cells which remain viable up to high NP concentration. Thus, these properties of the synthesized NPs were realized by reducing oxidative stress and inflammation and thereby reducing the atherogenicity.

## Author contributions

Conceptualization: SB, KA, NK; formal analysis: SB, HK, PK, NK, KA; funding acquisition; investigation: SB, HK, PK; project administration: NK, KA; supervision: NK, KA; validation: NK, KA; writing – original draft: SB; writing – review & editing: NK, KA.

## Conflicts of interest

There are no conflicts to declare.

## Supplementary Material

NA-004-D1NA00631B-s001

## References

[cit1] Daniel S. (2002). Nat. Med..

[cit2] Hennekens C. H., Gaziano J. M. (1993). Clin. Cardiol..

[cit3] Singh P. P., Mahadi F., Roy A., Sharma P. (2009). Indian J. Clin. Biochem..

[cit4] Mittal M., Siddiqui M. R., Tran K., Reddy S. P., Malik A. B. (2014). Antioxid. Redox Signaling.

[cit5] Grimsrud P. A., Xie H., Griffin T. J., Bernlohr D. A. (2008). J. Biol. Chem..

[cit6] Förstermann U., Xia N., Li H. (2017). Circ. Res..

[cit7] Münzel T., Camici G. G., Maack C., Bonetti N. R., Fuster V., Kovacic J. C. (2017). J. Am. Coll. Cardiol..

[cit8] Griendling K. K., Sorescu D., Lassègue B., Ushio-Fukai M. (2000). Arterioscler., Thromb., Vasc. Biol..

[cit9] Li H., Horke S., Förstermann U. (2014). Atherosclerosis.

[cit10] Wang Y., Subramanian M., Yurdagul A., Barbosa-Lorenzi V. C., Cai B., de Juan-Sanz J., Ryan T. A., Nomura M., Maxfield F. R., Tabas I. (2017). Cell.

[cit11] Shariat S. Z. A. S., Mostafavi S. A., Khakpour F. (2013). Iran. Biomed. J..

[cit12] He L., He T., Farrar S., Ji L., Liu T., Ma X. (2017). Cell. Physiol. Biochem..

[cit13] Asmis R., Jelk J. (2000). Arterioscler., Thromb., Vasc. Biol..

[cit14] Gotto A. M. (2003). J. Am. Coll. Cardiol..

[cit15] Salonen J. T., Nyyssönen K., Salonen R., Lakka H. M., Kaikkonen J., Porkkala-Sarataho E., Voutilainen S., Lakka T. A., Rissanen T., Leskinen L., Tuomainen T. P., Valkonen V. P., Ristonmaa U., Poulsen H. E. (2000). J. Intern. Med..

[cit16] Thomas S. R., Witting P. K., Stocker R. (1999). BioFactors.

[cit17] Jun Y., Shigehiro K., Takuro K., Toshiaki A. (1999). Atheroscler.

[cit18] Kim C. H. J., Mitchell J. B., Bursill C. A., Sowers A. L., Thetford A., Cook J. A., van Reyk D. M., Davies M. J. (2015). Atherosclerosis.

[cit19] Langbein H., Brunssen C., Hofmann A., Cimalla P., Brux M., Bornstein S. R., Deussen A., Koch E., Morawietz H. (2016). Eur. Heart J..

[cit20] Sugamura K., Keaney J. F. (2011). Free Radicals Biol. Med..

[cit21] Lewis D. R., Petersen L. K., York A. W., Zablocki K. R., Joseph L. B., Kholodovych V., Prud'Homme R. K., Uhrich K. E., Moghe P. V. (2015). Proc. Natl. Acad. Sci. U. S. A..

[cit22] Fredman G., Kamaly N., Spolitu S., Milton J., Ghorpade D., Chiasson R., Kuriakose G., Perretti M., Farokhzad O., Farokzhad O., Tabas I. (2015). Sci. Transl. Med..

[cit23] Wang Y., Li L., Zhao W., Dou Y., An H., Tao H., Xu X., Jia Y., Lu S., Zhang J., Hu H. (2018). ACS Nano.

[cit24] Gao Z., Horiguchi Y., Nakai K., Matsumura A., Suzuki M., Ono K., Nagasaki Y. (2016). Biomaterials.

[cit25] Yoshitomi T., Ozaki Y., Thangavel S., Nagasaki Y. (2013). J. Controlled Release.

[cit26] Sha S., Vong L. B., Chonpathompikunlert P., Yoshitomi T., Matsui H., Nagasaki Y. (2013). Biomaterials.

[cit27] Alaarg A., Pérez-Medina C., Metselaar J. M., Nahrendorf M., Fayad Z. A., Storm G., Mulder W. J. M. (2017). Adv. Drug Delivery Rev..

[cit28] Mulder W. J. M., Jaffer F. A., Fayad Z. A., Nahrendorf M. (2014). Sci. Transl. Med..

[cit29] Lobatto M. E., Calcagno C., Millon A., Senders M. L., Fay F., Robson P. M., Ramachandran S., Binderup T., Paridaans M. P. M., Sensarn S., Rogalla S., Gordon R. E., Cardoso L., Storm G., Metselaar J. M., Contag C. H., Stroes E. S. G., Fayad Z. A., Mulder W. J. M. (2015). ACS Nano.

[cit30] Wei X., Ying M., Dehaini D., Su Y., Kroll A. V., Zhou J., Gao W., Fang R. H., Chien S., Zhang L. (2018). ACS Nano.

[cit31] Dou Y., Chen Y., Zhang X., Xu X., Chen Y., Guo J., Zhang D., Wang R., Li X., Zhang J. (2017). Biomaterials.

[cit32] Katsuki S., Matoba T., Nakashiro S., Sato K., Koga J. I., Nakano K., Nakano Y., Egusa S., Sunagawa K., Egashira K. (2014). Circulation.

[cit33] Kheirolomoom A., Kim C. W., Seo J. W., Kumar S., Son D. J., Gagnon M. K. J., Ingham E. S., Ferrara K. W., Jo H. (2015). ACS Nano.

[cit34] Duivenvoorden R., Tang J., Cormode D. P., Mieszawska A. J., Izquierdo-Garcia D., Ozcan C., Otten M. J., Zaidi N., Lobatto M. E., Van Rijs S. M., Priem B., Kuan E. L., Martel C., Hewing B., Sager H., Nahrendorf M., Randolph G. J., Stroes E. S. G., Fuster V., Fisher E. A., Fayad Z. A., Mulder W. J. M. (2014). Nat. Commun..

[cit35] Basak S., Khare H. A., Roursgaard M., Kempen P. J., Lee J. H., Bazban-Shotorbani S., Kræmer M., Chernyy S., Andresen T. L., Almdal K., Kamaly N. (2021). Biomacromolecules.

[cit36] Li J., Zhang J., Chen Y., Kawazoe N., Chen G. (2017). ACS Appl. Mater. Interfaces.

[cit37] Garcia Y. J., Rodríguez-Malaver A. J., Peñaloza N. (2005). J. Neurosci. Methods.

[cit38] Palao-Suay R., Aguilar M. R., Parra-Ruiz F. J., Maji S., Hoogenboom R., Rohner N. A., Thomas S. N., San Román J. (2016). Polym. Chem..

[cit39] Warren N. J., Mykhaylyk O. O., Mahmood D., Ryan A. J., Armes S. P. (2014). J. Am. Chem. Soc..

[cit40] Zhang Y., Park A., Cintora A., McMillan S. R., Harmon N. J., Moehle A., Flatté M. E., Fuchs G. D., Ober C. K. (2017). J. Mater. Chem. C.

[cit41] Du J. Z., Tang L. Y., Song W. J., Shi Y., Wang J. (2009). Biomacromolecules.

[cit42] Li Z., Ma J., Lee N. S., Wooley K. L. (2011). J. Am. Chem. Soc..

[cit43] Zou J., Yu Y., Li Y., Ji W., Chen C. K., Law W. C., Prasad P. N., Cheng C. (2015). Biomater. Sci..

[cit44] Liu Z. H., Lv W. J., Zhao S. L., Shang Y. Z., Peng C. J., Wang H. L., Liu H. L. (2015). Comput. Condens. Matter.

[cit45] Gillich T., Acikgöz C., Isa L., Schlüter A. D., Spencer N. D., Textor M. (2013). ACS Nano.

[cit46] Łukasiewicz S., Szczepanowicz K., Błasiak E., Dziedzicka-Wasylewska M. (2015). Langmuir.

[cit47] Gofman J. W., Lindgren F., Elliott H., Mantz W., Strisower B., Herring V., Lyon T. P., Gofman J. W., Lindgren F., Elliott H., Mantz W., Hewitt J., Strisower B., Herring V. (1950). Science.

[cit48] Lynch S. M., Frei B. (1993). J. Lipid Res..

[cit49] Vazquez M. M., Gutierrez M. V., Salvatore S. R., Puiatti M., Dato V. A., Chiabrando G. A., Freeman B. A., Schopfer F. J., Bonacci G. (2020). Redox Biol..

[cit50] Chmielowski R. A., Abdelhamid D. S., Faig J. J., Petersen L. K., Gardner C. R., Uhrich K. E., Joseph L. B., Moghe P. V. (2017). Acta Biomater..

[cit51] Ma Y., Huang Z., Zhou Z., He X., Wang Y., Meng C., Huang G., Fang N. (2018). Free Radicals Biol. Med..

[cit52] Lodge J. K., Traber M. G., Sadler S. J. (2000). Lipids.

[cit53] Miura Y., Utsumi H., Hamada A. (1993). Arch. Biochem. Biophys..

[cit54] Lubos E. , Handy D. E. and Loscalzo J., Role of Oxidative Stress and Nitric Oxide in Atherothrombosis, 2008, vol. 13 10.2741/3084 PMC2617738 18508590

[cit55] He H., Oo T. L., Huang W., He L. F., Gu M. (2019). Sci. Rep..

[cit56] Singh U., Jialal I. (2006). Pathophysiology.

[cit57] Maurya R. P., Prajapat M. K., Singh V. P., Roy M., Todi R., Bosak S., Singh S. K., Chaudhary S., Kumar A., Morekar S. R. (2021). Clin. Ophthalmol..

[cit58] D'Ischia M., Palumbo A., Buzzo F. (2000). Nitric Oxide - Biol. Chem..

[cit59] Apak R., Güçlü K., Demirata B., Özyürek M., Çelik S. E., Bektaşoǧlu B., Berker K. I., Özyurt D. (2007). Molecules.

