# Supplementary material for: Nanoconfined anti-oxidizing RAFT nitroxide radical polymer for reduction of low-density lipoprotein oxidation and foam cell formation
Source: Nanoscale Adv. 2022 Jan 4;4(3):742–53. doi: 10.1039/d1na00631b (PMC9418007; doi:10.1039/d1na00631b)
Supplement: NA-004-D1NA00631B-s001 [file NA-004-D1NA00631B-s001.pdf]

Electronic Supplementary Material (ESI) for Nanoscale Advances.
This journal is © The Royal Society of Chemistry 2022

Supporting Information for

Nanoconfined anti-oxidizing RAFT nitroxide radical polymer for reduction of Low-Density Lipoprotein oxidation and Foam cell formation

Suman Basak[a,e], Harshvardhan Ajay Khare[a,b], Paul J. Kempen[a,c], Nazila Kamaly[d], Kristoffer Almdal[e]*

[a] *Department of Health Technology, DTU Health Tech, Technical University of Denmark, 2800, Kgs. Lyngby, Denmark*

[b] *Department of Clinical Physiology, Nuclear Medicine & PET and Cluster for Molecular Imaging, Rigshospitalet and University of Copenhagen, 2200, Copenhagen, Denmark*

[c] *National Centre for Nano Fabrication and Characterization, DTU Nanolab, Technical University of Denmark, 2800, Kgs. Lyngby, Denmark*

[d] *Department of Chemistry, Molecular Sciences Research Hub, Imperial College London, London, W12 0BZ, UK*

[e] *Department of Chemistry, Technical University of Denmark, 2800, Kgs. Lyngby, Denmark*

***Corresponding Author**

Email: kral@dtu.dk

Size exclusion chromatography (SEC)

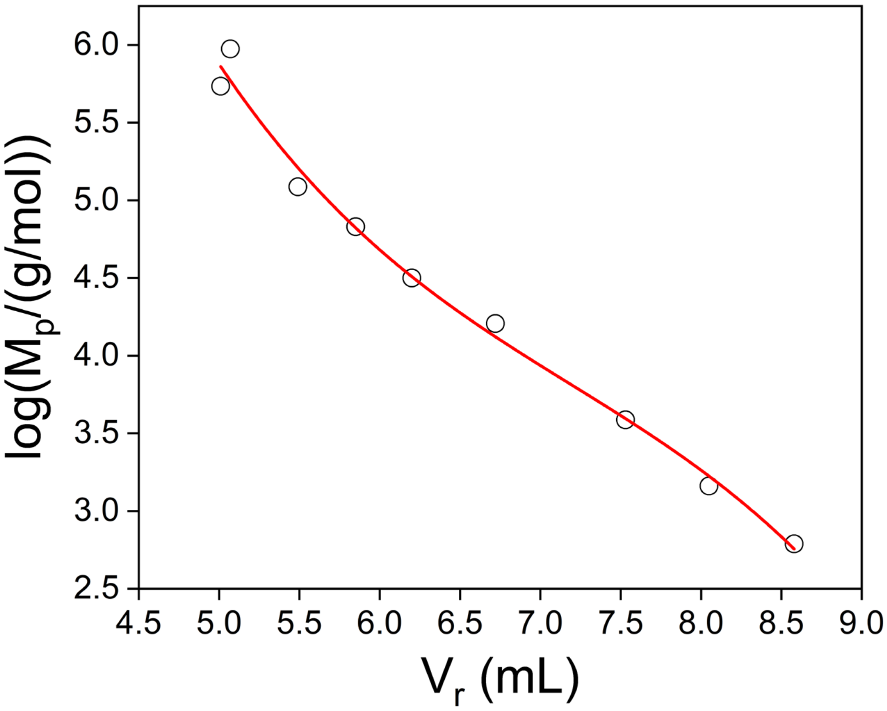

Fig. SI1 SEC PEG calibration. Eluent: HPLC-grade DMF, 50 mM LiCl, flow rate: 0.25 mL/min. Column: Mixed D, 300mm × 7.5mm. 30 min of equilibration time before each injection of 250 μL of sample. The red line is a 3rd order polynomial fit to the (V_r, log$M$) data points for polyethylene glycol (PEG) standards ($650 \leq M/\mathrm{Da} \leq 9.42 \times 10^5$).

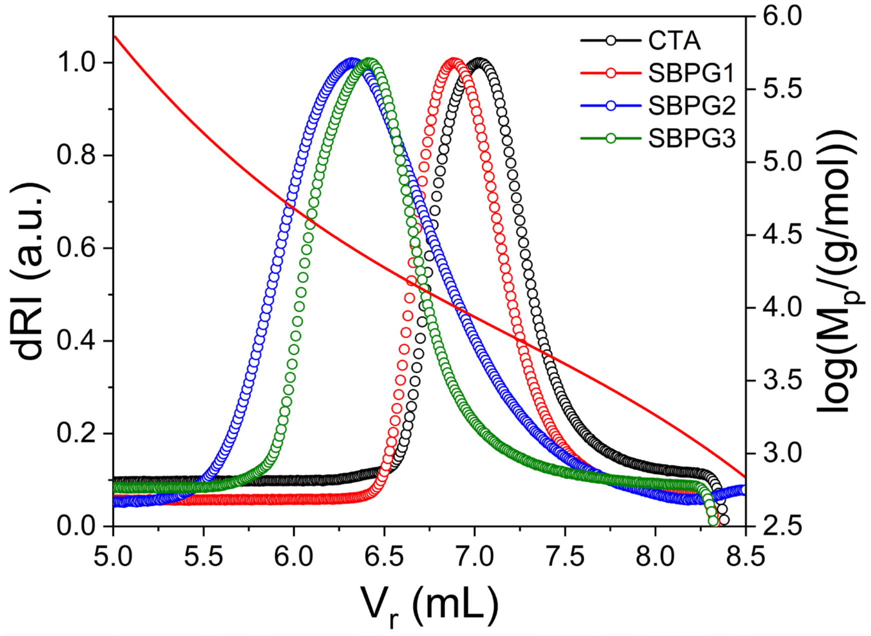

Fig. SI2 SEC of macro-CTA, SBPG1, SBPG2 and SBPG3 copolymer. Conditions as for Fig. SI1. The red line is the PEG standards based calibration curve - see Fig. SI1.

^{1}H-NMR characterization of SBPG1 and the macroinitiator macro-CTA

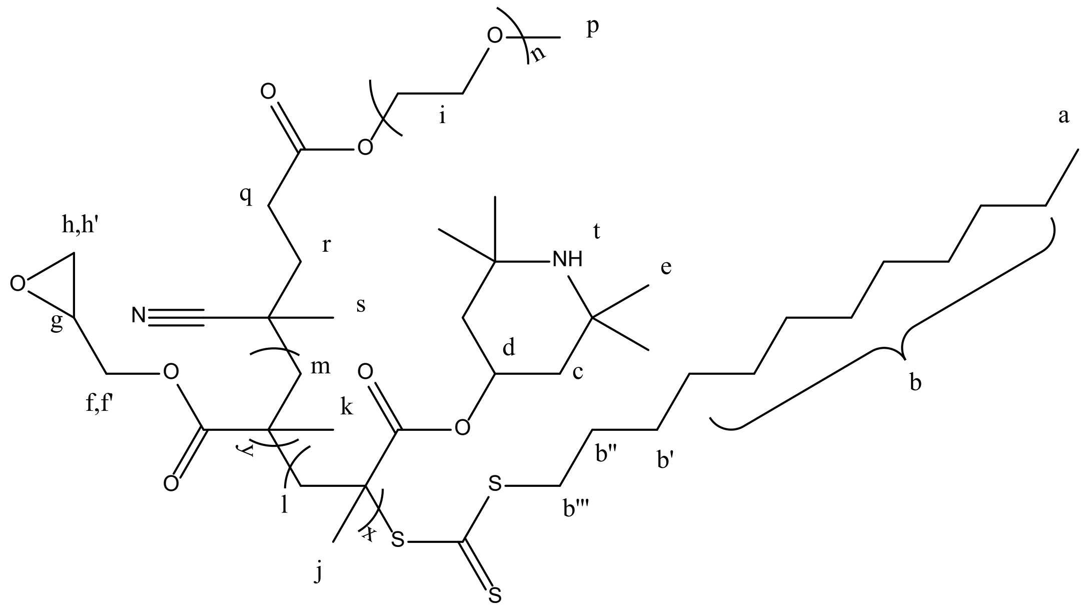

The NMR of the macro-CTA and sample SBPG1 are analysed based on the above proton labelling.

Fig. SI3 ^{1}H NMR assignments. The first CH_2-group in the polyethyleneglycol chain adjacent to the ester group have a distinct NMR signal for which the label i' is used. For sample macro-CTA x=y=0.

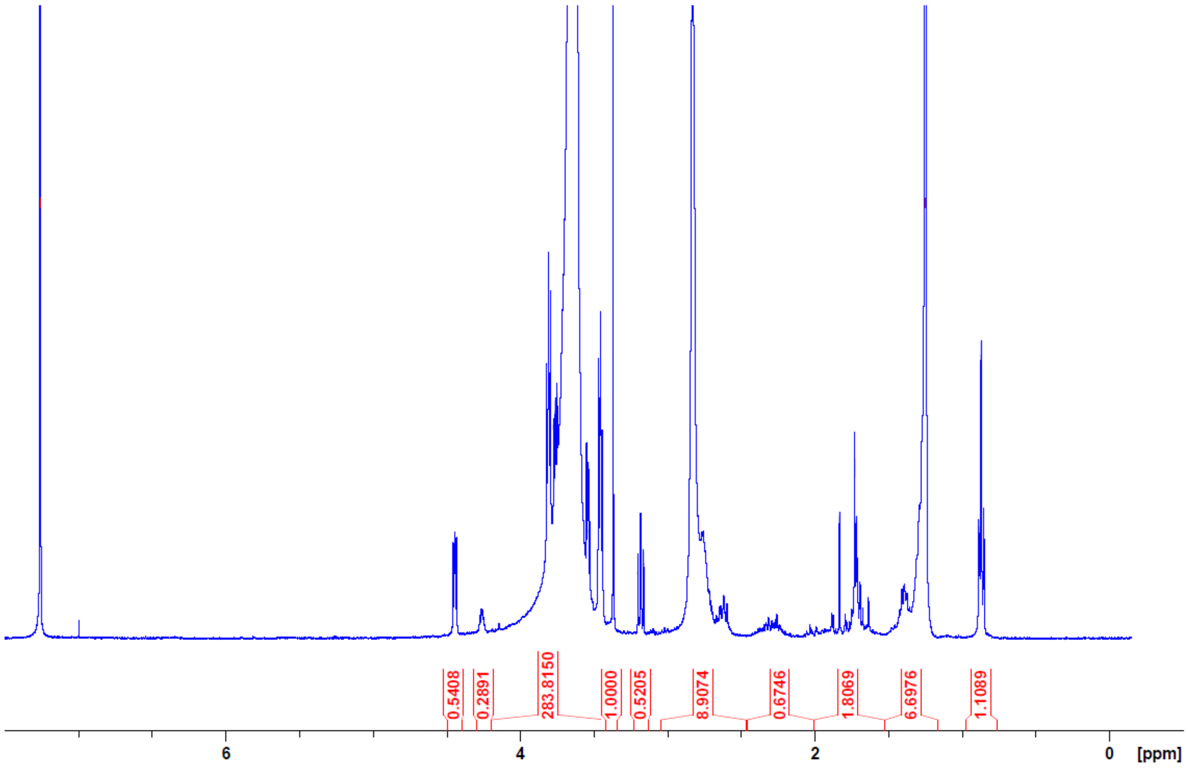

Fig. SI4 Macro-CTA ^{1}H NMR. Solvent: $CDCl_3$.

Table 1 NMR assignment for macro-CTA:

Integral	Range/(ppm)	value	Protons	# protons
1	0.765-0.977	1.1089	a	3
2	1.165-1.525	6.6976	b,b'	18
3	1.53-2.01	1.8069	b'',s	5
4	2.01-2.46	0.6746	r	2
5	2.47-3.05	8.9074	q	2
6	3.14-3.23	0.5205	b'''	2
7	3.34-3.42	1	p	3
8	3.42-.4.2	283.815	i	4n-2
9	4.2-4.3	0.2891	i'	2
10	4.4-4.5	0.5408		

This assignment fits the NMR-integrals with n=213.4. The largest discrepancies are: protons q only account for a minor part of integral 5. No protons are assigned to integral 10.

The molecular weight of the macro-CTA based on this analysis is:

$$M_{n,macroCTA} = 213.4 \times M_{\mathrm{EO}} + M_{\mathrm{C_{20}H_{38}S_3O_2N}} = 9.8\ \mathrm{kg/mol}.$$

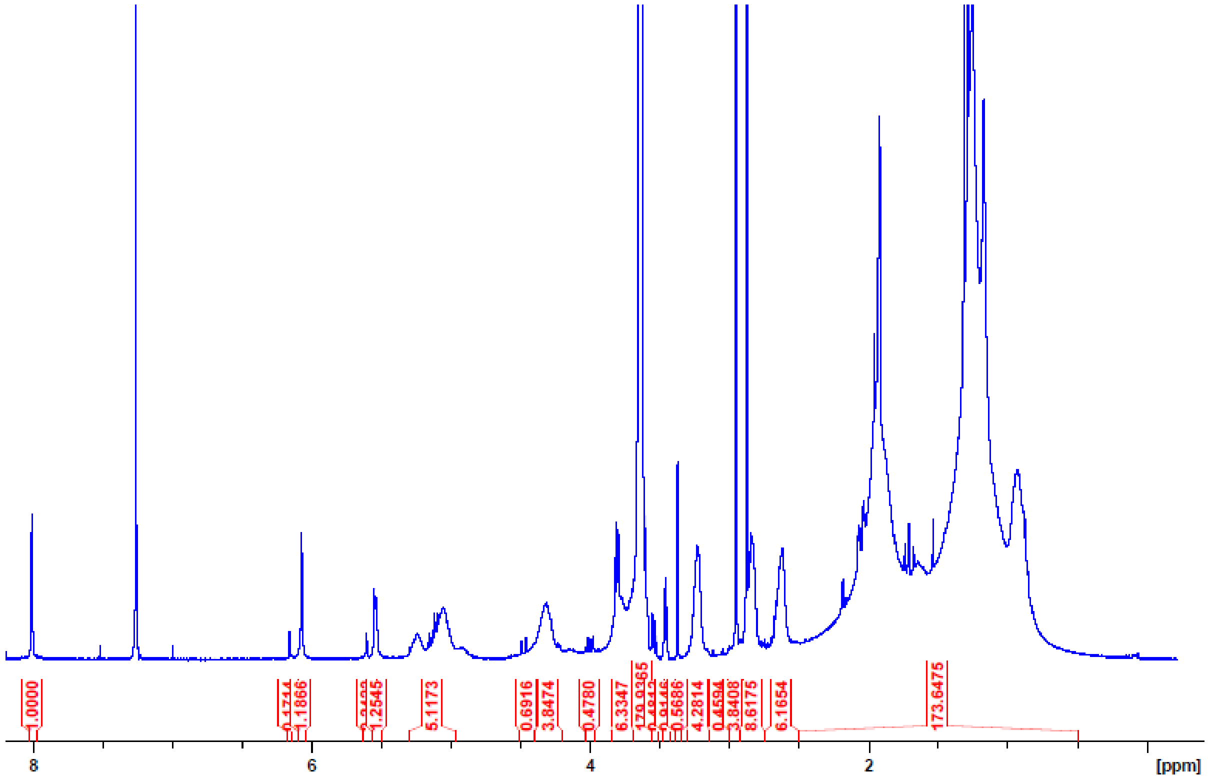

Fig. SI5 The NMR of SBPG1 copolymer. Solvent: $CDCl_3$.

Table SI2 NMR assignment for SBPG1:

Integral	Range/(ppm)	value	Protons	# protons
1	0.5-2.5	173.6475	a,b,b',b'',c,e,j,k,m,<br>s,r,t	19x+5y+28 and19 protons from PMA impurity
2	2.5-2.75<br>2.75-2.93<br>2.93-3.00<br>3.00-3.15<br>3.15-3.3<br>3.35-3.39<br>3.43-3.48<br>3.51-3.56<br>2.5-3.56	6.1654<br>8.6175<br>3.8408<br>0.4594<br>4.2814<br>0.5686<br>0.9146<br>0.4812<br>28.904783	b''',g,h,h',l,p,q,	2x+3y+7
3	3.56-3.69	179.9365	i	4n-2
4	3.69-3.85<br>3.97-4.04<br>4.2-4.4<br>4.4-4.51<br>3.69-4.51	6.3347<br>0.478<br>3.8474<br>0.6916<br>11.3517	f,f',i'	2y+2
5	4.97-5.3	5.1173	d	x and 1 proton from PMA impurity
6	5.5-5.57<br>5.57-5.63<br>6.05-6.1<br>6.15-6.18<br>5.5-6.18	1.2545<br>0.2422<br>1.1866<br>0.1714<br>5.1173	signal from PMA<br><br>signal from PMA	
7	7.98-8.03	1		

* The peak at 5.53 and 6.08 indicates that the NMR sample is not free from PMA. Integrals 1 and 5 are corrected for the PMA impurity. The corrected integrals can be fitted with n=213.4 (value obtained from the pure initiator) and x=y=27. The best fit is obtained by assuming that the pipiridinyl proton contributes to integral 1. Thus integral 7 is assigned to an unknown impurity. The fit overestimates integral 2 and 5 by 3.7 and 1.9, respectively. If the x=y assumption is released then x=27.4; y=25.2. Thus within error x=y.

The molecular weight of the SBPG1 is based on this analysis is:

$$M_{n,SBPG1} = 213.4 \times M_{\mathrm{EO}} + 27.2 \times (M_{\mathrm{GMA}} + M_{\mathrm{PMA}}) + M_{\mathrm{C_{20}H_{38}S_3O_2N}} = 19.8 \text{ kg/mol.}$$

Solubility characterization of copolymers.

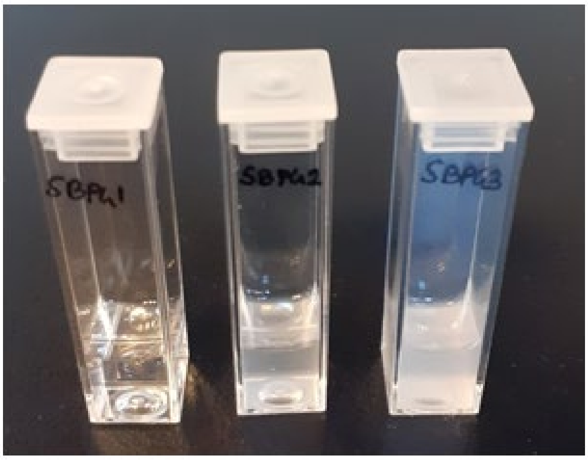

Fig. SI6 Solubility of copolymer SBPG1, SBPG2 and SBPG3 (from left to right) in DI water.

Oxidation of SBPG1

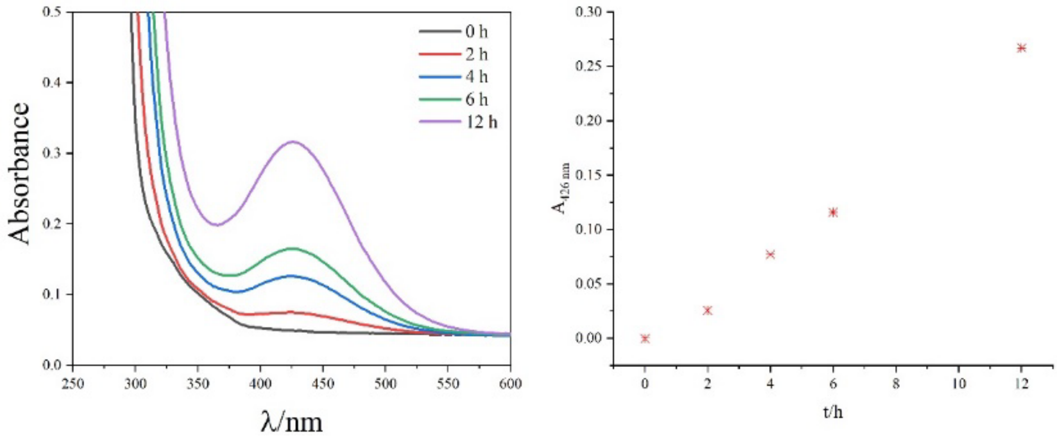

Fig. SI7 (left) Oxidation reaction of SBPG1 copolymer monitored by UV-spectrophotometry at different time frames. c_{SBPG1}=1mg/mL in dH_2O. (right) Absorbance at 426 nm using the t=0h as background.

The SBPG1 copolymer contains using the data above 21.5% (w/w) 2,2,6,6-tetramethyl 4-pipiridinyl-oxy units such that the 1mg/mL SBPG1 solution is 236 ppm in 4-oxy-2,2,6,6-tetramethyl 4-pipiridine-1-oxyl radical at full conversion. The solution of the SBPG copolymers are turbid, and thus

quantification of the degree of conversion is not easily possible. The turbidity enhances the absorption due to an increase in the path length of the light. The molar extinction of 4-hydroxy-2,2,6,6-tetramethyl-1-pipperidinyloxyl (TEMPOL) in dH_2O has been measured, $\epsilon_{TEMPOL} = 12.9M^{-1}cm^{-1}$. Using this value and the $A_{426\ nm}(12h)=0.2671$ lead to c_{TEMPOL} that is far above the theoretical. Thus substantial conversion was achieved.

Size characterization of SBPG1, 2 and 3 based micelles in DI water.

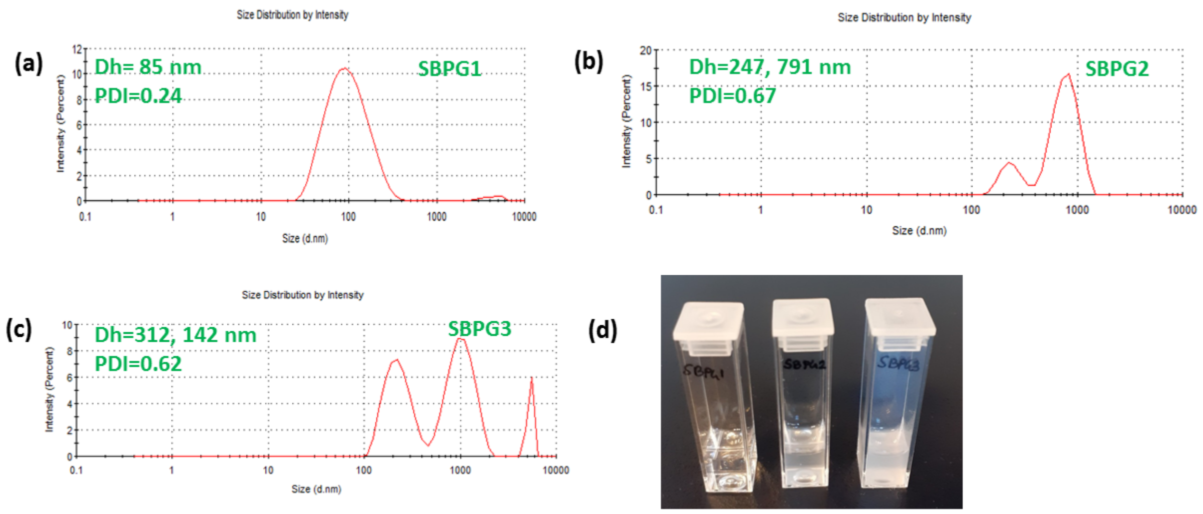

Fig. SI8 DLS (dynamic light scattering) (a) size of copolymer nanoparticles SBPG1. (b) SBPG2. (c) SBPG3.

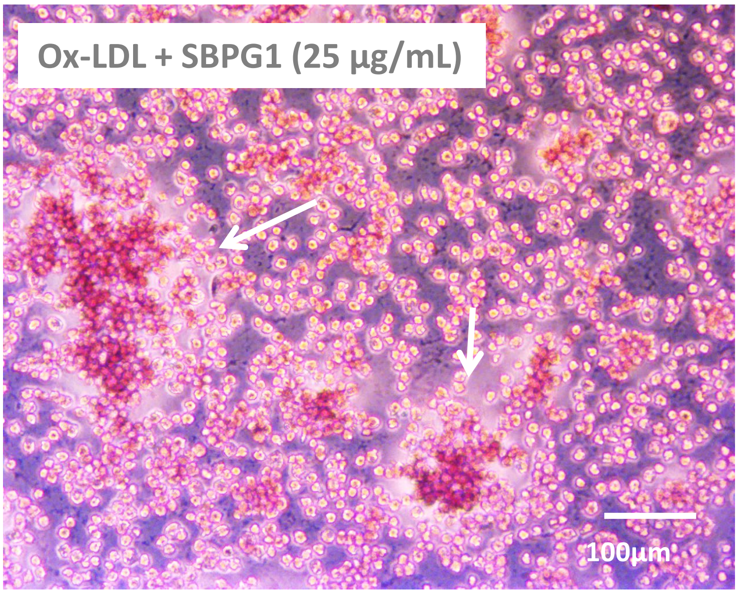

Fig. SI9 Oil red assay in RAW 264.7 cell lines. 50 µg/mL Ox-LDL; preincubated with SBPG1 NPs (white arrow indicates the oil droplet formation).

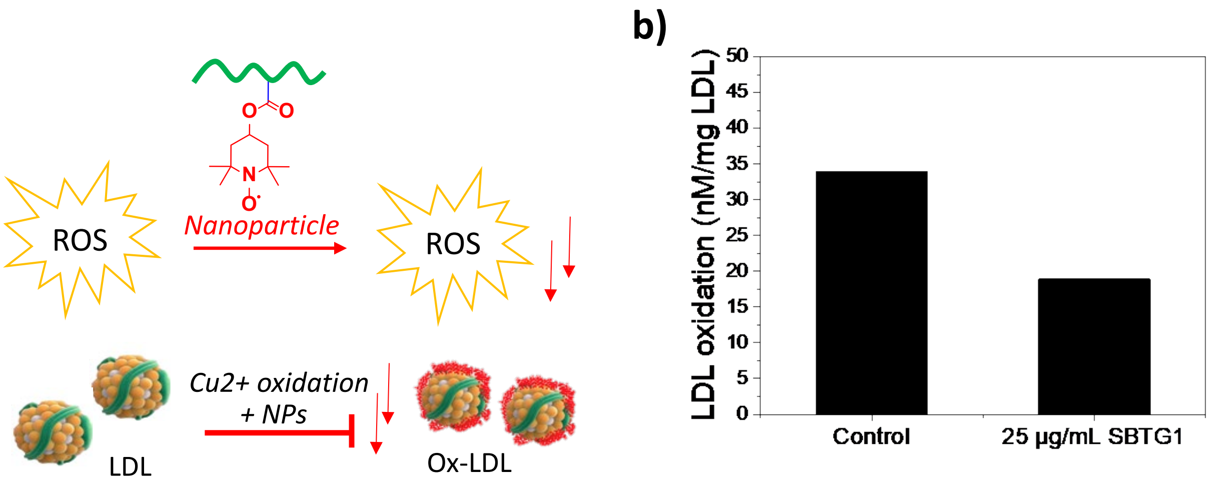

Fig. SI10 (a) Schematic illustration of RAFT NPs (SBTG1) reduce LDL oxidation. (b) Lipid Peroxidation (MDA) assay, pre-treated with RAFT nitroxide NPs. The data is the result of only two experiments where the error bar would be invisible on the graph.

The anti-ROS effectiveness of the RAFT nitroxide NPs was shown by the reduction of malondialdehyde **(MDA)** formation (Fig. SI10). MDA is the by-product of the arachidonate cycle and a principle aldehyde product of lipid peroxidation *in vivo*, that is being widely used as an indicator of oxidative **stress in biological systems**.[1] The **TBARS assay** is the simplest and most convenient method for quantifying lipid peroxidation in biological samples. The assay works on the reaction of TBA with MDA to produce a pink coloured MDA-(TBA2) Schiff base adduct.[1] TBARS assay has been applied in clinical studies linking oxidative stress response with cardiovascular risk. Therefore, we used LDL (100 µg/mL) in PBS buffer and preincubated it with and without addition of RAFT nitroxide NPs (e.g., SBTG1) for 60 min at room temperature. Then, 5 µM Cu^{2+} was added and incubated further for 4 h at 37 °C. The extent of oxidation was measured by the thiobarbituric acid **(TBA)** reactive substrate (TBARS) assay.

Total antioxidant capacity (TAC) can be attributed to single components in the defence systems against free radicals (Fig. 7b).[2] The measurement of the total oxidant status (TOS) accurately reflects the oxidative stress markers of atherosclerotic environment.[3,4] Oxidative stress occurs as a result of disruption of the balance between free radicals and the protective antioxidant system. This balance is expressed by the oxidative stress index (OSI), which is the ratio of total antioxidant status (TAS) to total oxidant status (TOS).[5]

References

1 K. Yagi, *Methods Mol. Biol.*, 1998, **108**, 101–106.

2 E. Romuk, C. Wojciechowska, W. Jacheć, J. Nowak, J. Niedziela, J. Malinowska-Borowska, A. Głogowska-Gruszka, E. Birkner and P. Rozentryt, *Oxid. Med. Cell. Longev.*, 2019, 7156038.

3 A. Sari, V. Davutoglu, E. Bozkurt, I. L. Taner and K. Erciyas, *Clin. Oral Investig.*, 2021, doi:10.1007/s00784-021-04144-8.

4 M. Karabacak, A. Dogan, S. Tayyar and H. A. Bas, *Med. Princ. Pract.*, 2014, **23**, 532–537.

5 U. Singh and I. Jialal, *Pathophysiology*, 2006, **13**, 129–142.
